# The Effect of* Morinda citrifolia* L. Fruit Juice on the Blood Sugar Level and Other Serum Parameters in Patients with Diabetes Type 2

**DOI:** 10.1155/2018/3565427

**Published:** 2018-08-06

**Authors:** Petra Algenstaedt, Alexandra Stumpenhagen, Johannes Westendorf

**Affiliations:** ^1^Department for Endocrinology and Diabetology, University Clinic Hamburg-Eppendorf, Martinistraße 52, D-20246 Hamburg, Germany; ^2^Institute of Experimental Pharmacology and Toxicology, University Clinic Hamburg-Eppendorf, Martinistraße 52, D-20246 Hamburg, Germany

## Abstract

**Background:**

The effect of the daily consumption of* Morinda citrifolia* (Noni) fruit juice on the physiological status of patients with diabetes type 2 (DT2) was tested over a period of two months.

**Methods:**

* Morinda citrifolia* (Noni) fruit juice (NFJ), 2 ml per kg bw per day, was consumed by twenty patients with DT2 after they underwent a standard treatment regimen including carbohydrate reduced diet and treatment with an antidiabetic drug and/or insulin. NFJ consumption started only after no further improvement was achieved. The intake of NFJ was terminated after eight weeks. The fasting blood sugar level was monitored every morning during the entire treatment period. Blood samples were taken before, at, and four and eight weeks after the start of NFJ intake. The analysis of the blood samples included the concentration of blood glucose, HbA1c, C-peptide, hs-CRP, triglycerides, total cholesterol, LDL, and HDL.

**Results:**

The consumption of NFJ by 20 patients with DT2 resulted in a significant mean decrease of the morning blood sugar level monitored over a period of eight weeks. While NFJ reduced the blood glucose level in several but not all hyperglycemic patients, it did not cause hypoglycemia in normoglycemic patients. NFJ consumption also reduced the mean HbA1c value significantly (p= 0.033). Significant decreases (p= 0.01) were also achieved for high sensitive CRP values in patients starting with high levels (>2 mg/L), whereas no change was observed in patients with normal values (< 2 mg/L). The level of C-peptide showed a significant mean increase after four weeks of NFJ consumption in those patients who started with low levels (<3 *μ*g/l, p=0.004, N=11) but not in patients with higher levels (> 3 *μ*g/L).

**Conclusion:**

The daily consumption of NFJ has the potential to regulate elevated blood sugar levels and some other pathological parameters in patients with DT2. NFJ therefore serves as a suitable additive to the diet of diabetic patients.

## 1. Background

Diabetes type 2 (DT2) is a metabolic disorder characterized by high blood glucose levels, insulin resistance, and a relative lack of insulin. The global number of patients diagnosed with DT2 has increased from 30 million in 1985 to 392 million in 2015 [1]. Obesity and lack of exercise significantly enhance the risk of developing DT2, which shows a higher incidence in industrial compared to underdeveloped countries [2]. DT2 is associated with increased incidences of secondary diseases, such as arteriosclerosis leading to strokes, ischemic heart disease and lower limb amputations, reduction of glomerular filtration and kidney failure, and retinopathy leading to blindness [1, 3].

The primary goal of DT2 management is to reduce the fasting blood sugar level to < 130 mg/dl. Because of the high uncertainty of the daily fasting blood glucose level, the HbA1c value is preferentially used to characterize the glycemic status of patients with diabetes [4]. This value represents the percentage of glycosylated hemoglobin accumulated during the previous 8-12 weeks and is less sensitive to deviations. Healthy patients have values around 5%. A goal of the clinical diabetes management is to keep the HbA1c level below 7%. This is facilitated by weight reduction, a diet poor in carbohydrates, and regular exercise. Additionally, antidiabetic drugs or insulin may be necessary to adjust the blood glucose and HbA1c level. These drugs act on either the intestinal glucose absorption, biliary gluconeogenesis, insulin production and secretion, or the transcellular glucose transport. Antidiabetic drugs have several side effects, like gastrointestinal and metabolic disturbances, hypoglycemic sensations, increased risk of cardiovascular disease, and others [5].

Long before the availability of synthetic drugs, plants and their products have been used to treat diabetes. Today treatment of diabetes with phytomedicine is mostly used in underdeveloped countries, where access to synthetic drugs and insulin is limited, or in industrialized countries with tradition in the use of alternative medicine, such as China and India [6–10]. Although alternative treatment of diabetes is rarely taught in medical schools or practiced in hospitals, an increasing number of patients in Europe, USA, and Canada use nonprescribed remedies to treat their disease, mostly in addition to the conventional therapy [11–14]. Although hundreds of plants and plant products have been identified as useful in the treatment of diabetes in traditional medicine, only a few have undergone systematic investigation to demonstrate effectiveness [15]. Mechanisms of improving insulin resistance, enhancement of *β*-cell function, suppression of appetite, stimulation of energy expenditure, and regulation of lipid and carbohydrate metabolism have been shown for some plant derived antidiabetic remedies mainly from animal experiments [15–18]. Clinical trials to support the findings obtained from animal experiments are rare.

The fruits and leaves of the tropical plant* Morinda citrifolia *L. (Noni) have been used as a food and medicine by indigenous people of South East Asia and the Polynesian Triangle for hundreds of years. Noni was one of the most important medicinal plants for Polynesian people, who used it for multiple reasons, among which was also the treatment of diabetes [19, 20]. In the last twenty years, Noni fruit juice (NFJ) became popular worldwide as a wellness drink with the European Union authorizing the sale of NFJ in 2003 under the Novel Food Regulation [21]. Although among the benefits reported by consumers an improvement in the management of diabetes is included [22], systematic clinical trials to support the consumer testimonies are lacking. Our knowledge about the antidiabetic activity of extracts of Noni fruits and leaves comes from investigations in animal and tissue culture models for diabetes, which demonstrated an insulin-like enhancement of glucose uptake in fat and muscle cells [23].

The present clinical investigation was performed to demonstrate a possible antidiabetic action of NFJ in patients with DT2.

## 2. Methods

### 2.1. Patients

Twenty patients of the Endocrinological Department of the University Clinic Hamburg-Eppendorf diagnosed with DT2 participated in the investigation. Age, gender, fasting blood glucose level, HbA1c-value at the start of the observation period, and medication are listed in Table 1. All patients had undergone a standard management program for their condition independently from the study in order to optimize their glycemic status, before entering the study. This included adaptation of the diet, physical exercise, and medication where necessary. Patients were given the opportunity to participate in the investigation after no further improvement by the standard treatment was achieved. Patients with a poor overall health status or those with doubt about compliance were not selected to participate in the study.

### 2.2. Intake of NFJ and Collection of Data

NFJ is an authorized novel food in the* European Union* that confirmed that its consumption is not associated with a health risk. The clinical management of the patients attending the study did not differ from the regular schedule of patients with diabetes in the facility, which excluded any additional risk caused by the participation in the study. The data taken were a normal part of the clinical management of patients with diabetes. A special authorization by an ethical committee was not needed, because the law of the State of Hamburg, Germany, allows the use of anonymized data of patients for research purposes [24]. Nevertheless, the protocol strictly followed the principles laid down in the declaration of Helsinki [25]. All patients involved in the study were informed about the scientific background of the study, the procedure of collecting data, the anonymization, and the use of the data. A written agreement of the patients was obtained prior to participation.

NFJ (Tahitian Noni™ Juice) was kindly provided by the Company Morinda Inc., Provo, Utah, USA. The juice consisted of 89% fermented Noni fruit puree harvested in French Polynesia, blended with 11% of blueberry and red grape juice, to mask the unpleasant taste of the Noni fruits. Patients were asked to drink 2 ml NFJ/kg bw per day over a period of eight weeks. Blood samples were taken before and four and eight weeks after the start of NFJ consumption. The blood was analyzed by a routine screening protocol used in the facility for 67 different parameters like blood cells, electrolytes, liver and kidney parameters, hormones, and others (e.g., HbA1c, ferritin, and interleukins). Fasting blood glucose levels were monitored by the patients daily or every second day during the period of the NFJ consumption.

### 2.3. Statistical Analysis

All calculations of means and standard deviations, p values, and linear regression curves were performed by using the OriginPro® 9.1 Data Analysis and Graphing Software of Origin Lab® Corporation, Northampton, MA, USA, as well as Microsoft Excel®, 2010. p values of changes in HbA1c, C-peptide, and hs-CRP blood levels were calculated from paired t-test (values of day 0 against values after four or eight weeks). The p value of the slope of linear regression curves of fasting blood sugar values of patients between days 1 and 50 of the intake of NFJ was calculated by the one sample t-test.

## 3. Results

**Figure 1:**
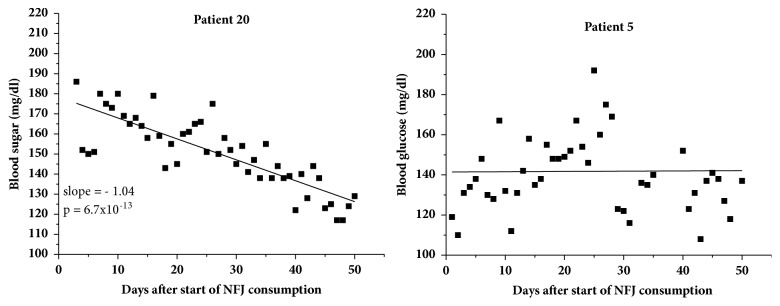
Fasting blood sugar level of two patients during 50 days of NFJ intake (2 ml/kg bw per day). p value for patient 20 was calculated from t-test of the linear regression coefficient.

Fasting blood sugar profiles monitored over the entire period of NFJ consumption were available for 19 out of 20 patients. One patient (no. 16) reported only a limited number of blood sugar values and was excluded from the calculation. As expected, the daily blood sugar values showed considerable variation, which made statistical calculations difficult. Linear regression curves were therefore calculated for each individual blood sugar profile between the start and termination of the NFJ consumption. Two examples are shown in Figure 1. Patient 20 experienced a continual decrease of his fasting blood sugar value after the start of the NFJ consumption of about 50 mg/dl although the triple medication before was not able to reduce the blood sugar level below approx. 180 mg/dl. In contrast, patient 5 did not respond at all. The results of all patients are shown in Table 2. The “*intersection with ordinate*” at day zero and day 50 represents the values of the blood sugar levels at the beginning and termination of the NFJ consumption, extrapolated from the linear regression of the blood sugar curves. This procedure reduces the spontaneous daily deviations in the blood sugar level. Negative slopes of the linear regression curves of the blood sugar level, indicating a decrease after NFJ consumption, were observed in 14 out of 20 patients. The mean and SD of the initial blood sugar value calculated from the regression curves were 139 ± 23. The corresponding values for the final blood sugar values were 125 ± 22 mg/dl. A paired t-test performed with the individual differences between the initial and final blood sugar values revealed a significant result with a p value of 0.0024. The mean and SD of the slopes of the linear regression of the blood sugar curves of the patients were -0.28  ±  0.34 mg/dl per day with a p value of 0.002, calculated from a one sample t-test. HbA1c levels of the patients under consideration at the start of the NFJ intake are shown in Table 3. All patients underwent a diabetes management program prior to the start of NFJ intake which resulted in HbA1c levels in the range of 5.8–7.9% for those recruited in the study. A significant reduction (p= 0.033) of the mean HbA1c-value of 0.27 ± 0.5% was achieved after 8 weeks of NFJ consumption on top of the routine clinical diabetes management of the patients.

**Figure 2:**
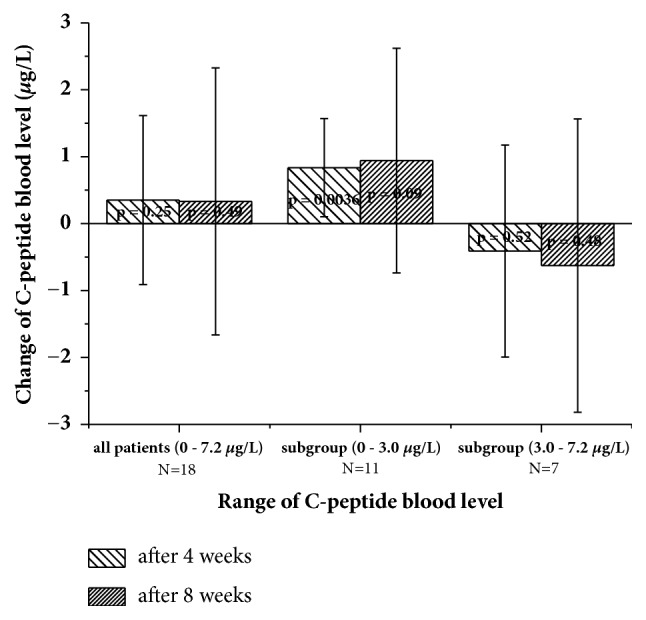
Changes in C-peptide blood level after four and eight weeks of NFJ consumption (2 ml/kg bw and day). p values were calculated from paired t-test (values at start against values after 4 or 8 weeks).

The blood concentration of C-peptide is used as an indicator of the ability of pancreatic beta-cells to excrete insulin. C-peptide is liberated during transformation of proinsulin to insulin and excreted together with insulin in equimolar amounts. Its concentration is considerably more stable in blood compared to insulin, which makes it a preferable parameter for monitoring insulin secretion. Data of C-peptide blood concentrations were available for 18 patients. Because consumption of carbohydrates will cause an elevation of the C-peptide level lasting up to two hours, all patients agreed not to eat for two hours prior to the blood collection. Eleven out of 18 patients had normal or reduced ranges of C-peptide between 0.7 and 3.0 *μ*g/L at the start of the NFJ consumption. Seven patients had elevated levels of 3.1-7.2 *μ*g/L. Of the 11 patients with normal or reduced ranges of C-peptide, 10 showed an increase and six of the seven patients with elevated levels > 3 *μ*g/L showed a decrease after four weeks of NFJ consumption. Considering all patients, the mean increase after four and eight weeks was 0.35 ± 1.26 (p=0.25) and 0.33 ± 1.99 (p=0.49) *μ*g/L, respectively, and thus not significant. Different results were achieved if the patients are split into groups with normal or reduced C-peptide levels (0.7 – 3.0 *μ*g/L) and those with elevated levels (3.1–7.2 *μ*g/L). The results are demonstrated in Figure 2. Significant increases were obtained at normal or reduced C-peptide range between 0.7 and 3.0 *μ*g/L (p= 0.0036) after four weeks of NFJ consumption. Although the absolute values of the increases in C-peptide levels are greater after eight compared to four weeks, the statistical significance is lower because of an increased variation within this group. DT2 is associated with an elevated risk of complications in the cardiac circulatory system. Control of the blood lipid values is therefore an important part of the clinical management of the disease. We therefore analyzed the blood concentrations of triglycerides and total cholesterol as well as HDL and LDL concentrations during the observation period. The level of triglycerides of the patients before the intake of NFJ showed considerable variation (254 ± 172 mg/dl). The lowest concentration (patient 3) was 60 and the highest (patient 14) was 580 mg/dl. Eleven out of 20 patients had values > 200 mg/dl. Independently of the starting level, some patients showed an increase and others a decrease of the triglyceride blood level after four and eight weeks of NFJ consumption. A clear trend was not observable.

Five out of 19 patients had total cholesterol levels >200 mg/dl at the start of the NFJ consumption. After eight weeks the mean blood level of these patients was reduced from 258 ± 50 mg/dl to 248 ± 43 mg/dl (p= 0.07).

Calculations of statistics for LDL-levels interfered with high triglyceride levels. There were ten triglyceride levels > 400 mg/dl in five patients, which did not allow estimation of LDL-levels. Another six vales were between 300 and 400 mg/dl, making the estimated LDL values questionable. Because of the reduced available LDL blood levels, no statistical calculation was performed.

The mean of HDL-levels of the patients at the start of the NFJ intake was 41.0 ± 10.6 mg/dl. The corresponding values after four and eight weeks of NFJ intake were 42.3 ± 10.3 mg/dl and 41.75 mg/dl, respectively. The differences were not statistically significant.

**Figure 3:**
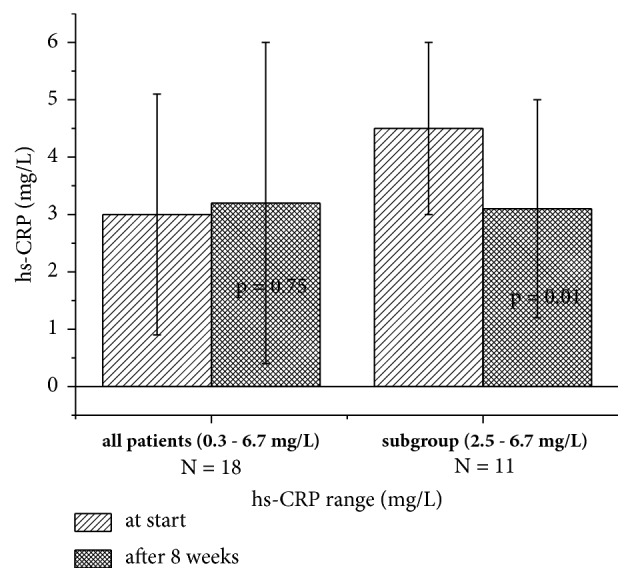
hs-CRP values of patients at the start and after consumption of NFJ (2 ml/kg BW per day). p values were calculated from paired t-test (p values at start against values after 4 or 8 weeks).

The blood level of high sensitive C-reactive protein (hs-CRP) is used as a marker for the risk of complications arising from CVD, like heart attack, strokes, and others. There was a consensus that values > 5 mg/L indicate a high risk; however, more recent studies suggest that this might be the case already with levels > 2 mg/L [26]. hs-CRP levels were available for 18 out of 20 patients. Of these, four had values > 5 (5.2–6.7) mg/L and eleven had values > 2 (2.5–6.7) mg/L. The development of the hs-CRP values during the consumption period of NFJ is shown in Figure 3. No significant changes of the mean values after eight weeks of NFJ consumption are seen, if all patients are included in the calculation; however, if only patients with values > 2 mg/L are considered, a highly significant decrease occurs after eight weeks (p=0.01).

## 4. Discussion

DT2 is one of the greatest health threats in modern societies, potentially leading to morbidity and early death. The disease is closely related to excess nutrition and lack of physical exercise. Consequently, control of nutrition and enhancement of physical exercise are primary mechanisms of diabetes management; however these actions are not sufficient in many cases, especially for those in advanced stages of the disease. Most patients with DT2 receive additionally an oral medication and/or injection of insulin [27]. A variety of synthetic drugs with hypoglycemic activities are used. The drug categories include sulfonylureas, biguanides, alpha-glucosidase inhibitors, thiazolidinediones, and meglitinides. Their mechanisms of action involve the enhancement of insulin secretion, the improvement of insulin sensitivity in muscle and liver cells, the inhibition of hepatic glucose formation, enhancement of muscle glucose uptake, and the inhibition of carbohydrate digestion [15]. Each of the different mechanisms has its own side effects, which can be mild, such as gastrointestinal disturbances, or severe, such as liver and kidney toxicity.

Phytotherapy is an alternative to the treatment of diabetes with synthetic drugs, mainly used in countries like China and India; however, its acceptance in Europe and North America is increasing. Although several hundred plants have been reported to be useful in the treatment of diabetes, in most cases a systematic scientific evaluation of their effects is lacking [15]. Further investigation of traditional medicinal herbs useful in diabetes treatment is recommended by the WHO Expert Committee on diabetes [13].

Similar to synthetic drugs, plants with hypoglycemic properties act also via different mechanisms, among which are the inhibition of carbohydrate digestion and glucose absorption, enhancement of glucose uptake into liver and muscle cells via upregulation of glucose transporters, regulation of fatty acid, carbohydrate, and glucose metabolism via activation of nuclear receptor PPAR-*γ*, and insulinomimetic and insulinotropic effects [19]. Most of the plants used for the treatment of DT2 do not have the side effects of synthetic drugs. Moreover, antidiabetic phytomedicines often have additional activities, which are directed against the long term collateral damage caused by DT2 [15]. This is possible, because plants and herbal extracts contain a variety of compounds with antioxidative, anti-inflammatory, wound healing, antimicrobial, and hormone regulating properties, which act on the complex clinical symptoms associated with diabetes mellitus.

Noni plants have been used by indigenous Polynesian people for the treatment of diabetes for hundreds of years [20, 21]. After 1996, NFJ became very popular as a wellness drink worldwide, but also for relief of inflammatory pain, to improve the immune defense system and physical endurance and also to lower the blood glucose level and adverse symptoms associated with diabetes [23]. Our knowledge about the antidiabetic activity of NFJ comes mainly from preclinical investigations [28–31]. Nayak et al. [28] have shown that a NFJ dose of 2 ml/kg bw twice a day decreased the fasting blood glucose level in streptozotocin induced diabetic rats to 150 mg/dl compared to 300 mg/dl in controls. A marked decrease of the blood glucose and HbA1c levels was also observed in diabetic mice fed with fermented Noni fruit juice [30]. The authors of this study also demonstrated that fermented NFJ stimulated the glucose uptake into cultured C2C12 cells via stimulation of AMP-activated protein kinase.

In our clinical study, 20 patients consumed 2 ml/kg bw of NFJ once a day. In contrast to the rat experiment, NFJ was taken by the patients additionally to the standard therapy including adaptation of the diet, enforcement of physical exercise, and application of antidiabetic drugs. Nevertheless, significant decreases of the blood glucose levels and HbA1c were observed. Patient 20, who received a triple medication with metformin, insulin, and a GLP1-receptor antagonist, experienced an additional decrease of the blood sugar level of about 50 mg/dl during the intake of NFJ. The mean and SD of the slopes of the blood sugar curves of the patients between start and termination of NFJ consumption after eight weeks were -0.28 ± 0.34 mg/dl per day with a p value of 0.002. Thus, the decrease of the blood sugar values was highly significant. This was also confirmed by the comparison of the HbA1c values between start and termination of the NFJ consumption. A mean decrease of 0.2 ± 0.7% was achieved after eight weeks (p value = 0.03). Although this decrease was only small, it should be noted that it is significant and on top of a standard management of diabetes. Moreover, the observation time of eight weeks is the minimum duration for changes of the HbA1c-value to be detectable. Thus, the clinical data from the present investigation are in line with the experimental data obtained previously with laboratory animals.

Mechanistic experiments regarding the hypoglycemic effects of NFJ have been performed in animals and with tissue culture [28–31]. Such results demonstrated that NFJ exerts an insulin mimetic activity. Applied together with insulin, NFJ synergistically improved the hypoglycemic effect of insulin in alloxan-induced diabetic rats [31]. Insulin mimetic effects of NFJ were also demonstrated in 3T3-L1 adipocytes; however, no synergistic activity with insulin was observed [30]. Activation of AMP-kinase (AMPK) via phosphorylation leads to an increased uptake of glucose in adipocytes and muscle cells [32]. Lee et al. [30] demonstrated that fermented NFJ increased the phosphorylation of AMPK in C2C12-derived myotubes in culture in a dose dependent manner. Furthermore, the authors showed that NFJ also enhanced the activity of PPAR-*γ*, a transcription factor that regulates gene expression in the liver, adipose tissue, vascular endothelium, and muscle. Activation of PPAR-*γ* increases the expression of the glucose transporter GLUT-4 and its translocation into the cell membrane of adipocytes [33]. It also decreases the output of glucose from liver cells [34]. It is therefore likely that the improvement of the glucose homeostasis observed in our study with DT2 patients is at least partly attributed to an increase of an insulin-independent uptake of glucose into energy storage cells and a decrease of the output from liver cells. Our finding that the C-peptide secretion is increased after consumption of NFJ in patients with a low blood level (< 3 *μ*g/L) but not in patients with higher levels (3-7 *μ*g/L) additionally indicates that NFJ may also be able to restore the feedback of the glucose blood level on the insulin synthesis in and/or secretion from pancreatic *β*-cells. Thus, patients with DT2 may benefit from two different mechanisms of NFJ, one of which is directed to the glucose storage cells and the other to insulin supply.

Diabetes is associated with a variety of secondary consequences, which increase the morbidity and reduce the life expectancy. Most critical ones are micro- and macrovascular damage, leading to ischemic heart disease, stroke, kidney failure, blindness, and lower limp amputations [1]. Disturbances of the lipid metabolism with elevated cholesterol levels and inflammatory processes are involved in the damage of vascular tissue [33]. Synthetic drugs used for the treatment of DT2 reduce the blood sugar level; however, they have no or little influence on the complications associated with the disease. Plant derived preparations are complex in composition and often contain compounds with multiple benefits for patients with diabetes [6, 10, 16, 17, 19, 20]. This is also the case for NFJ, which is known to stabilize and restore the homeostasis. Epidemiological surveys among consumers have shown that regular consumption of NFJ is able to enhance the energy and physical endurance, improve inflammatory pain in joints and back, and strengthen the immune system [23, 35]. A double-blind placebo controlled clinical trial among heavy smokers has shown that NFJ is able to reduce the cholesterol level significantly [36]. A differential analysis of the serum lipid fraction made in this trial showed a decrease of triglycerides and LDL and an increase of HDL. An improvement of the blood cholesterol status of patients with DT2 was also observed in our study. A small nonsignificant reduction of 3 and 4 mg/dl total cholesterol was observed after four and eight weeks of NFJ consumption, respectively. If only patients with elevated cholesterol levels (> 200 mg/dl) were considered (N=5) the reduction after four and eight weeks was 15 and 17 mg/dl. Statistical calculations of LDL-levels were not possible, because several patients had triglyceride levels of > 400 mg/dl, which made monitoring of the LDL-levels difficult or impossible. Triglyceride levels of patients were reduced by NFJ intake in some cases and elevated in others. A clear trend was not observable.

Inflammatory processes are involved in obesity and DT2. Reduction of inflammation markers, such as cytokines (IL6, TNF-*α*) and CRP may lead to an improvement of glycaemia and cardiovascular prognosis [37]. One of the most beneficial properties of NFJ is its anti-inflammatory activity, which has been proven in epidemiological investigations [23, 35] and preclinical studies in tissue culture [38] and animal models [39, 40]. Recently we observed anti-inflammatory effects in dentistry patients with gingivitis after mouth wash with NFJ followed by swallowing [41]. In the present study we investigated the effect of NFJ on the inflammation marker hs-CRP of patients with DT2. Eleven out of 19 patients had elevated blood levels > 2 mg/L. After eight weeks, the mean hs-CRP values of these patients were reduced from 4.5 ± 1.5 to 3.1  ±  1.9 mg/L. This change was highly significant (p = 0.01).

NFJ contains numerous compounds with beneficial health effects. Among these are flavonoids (quercetin, kaempferol), coumarins (scopoletin), iridoids (deacetylasperulosidic acid, asperulosidic acid, and asperuloside), and others [42–45], which have been demonstrated to exert antioxidative and anti-inflammatory properties [46–49]. Special attention has been given to the iridoid fraction in NFJ because of the biological activity profile of this group of compounds [50]. The most abundant iridoid in NFJ is deacetylasperulosidic acid (DAA) [45]. This compound has been shown to exert antioxidative and anti-inflammatory properties in rats [51]. Monotropein, an iridoid which is closely structurally related to DAA, also showed antinociceptive and anti-inflammatory activity in rats [52]. We have recently demonstrated that DAA is absorbed and excreted unchanged after oral application to mice [53]. This finding was unexpected because DAA is an iridoid glucoside, which is expected to be hydrolyzed by intestinal and/or liver glucosidases. Plants synthesize iridoid glycosides for defense purposes against herbivorous predators [54]. The aglycones released after hydrolysis are highly reactive and bind to bioactive functional proteins of the intestinal wall and liver, which are inactivated. The lack of sensitivity of murine (and other mammalian) glucosidases towards DAA is most probably an evolutionary adaptation of the animals to bypass the toxicity of DAA and similar compounds. We also found that some other iridoids, structurally related to DAA (monotropein, geniposidic acid, and asperulosidic acid), appeared unchanged in the urine after oral application to mice (unpublished results). The systemic appearance of iridoid glycosides, which are bitter in taste, could nevertheless be recognized as potential toxic via bitter taste receptors [55]. Such receptors (i.e., T2Rs) are distributed in several organs and initiate a cascade of defense mechanisms in order to combat with the impact of the expected toxicity [56]. T2R16 has been demonstrated to be sensitive to *β*-glucopyranosides, the chemical group to which DAA and other iridoids belong [57]. Properties of iridoids, such as an enhancement of the antioxidative potential, decrease of inflammatory processes, pain perception, and metabolic effects, could be interpreted as an adaptogenic response to potential toxic compounds with bitter taste. T2Rs present in some gastrointestinal cells have been shown to secrete the peptide hormones ghrelin and glucagon-like peptide-1 (GLP-1) in response to stimulation by bitter tasting compounds [56]. GLP-1 stimulates the excretion of insulin in response to an increase of the blood glucose level [58]. This mechanism could also be involved in the observed effects of NFJ on the blood glucose level of patients with DT2 involved in our study. The fact that some of the patients showed a response of the blood glucose level after NFJ consumption, while others did not, is possibly due to individual differences in the expression of extraoral bitter taste receptors, which show an extensive polymorphism within the human population [59]. The widespread distribution of iridoids in plants used as food and spices could open new strategies in the treatment of diabetes and other diseases; however, the validity of this hypothesis has to be confirmed by further investigations.

## 5. Conclusion

The present pilot study showed that the consumption of 2 ml/kg bw of NFJ over a period of eight weeks caused a significant decrease in fasting blood glucose levels and HbA1c values in twenty patients with diabetes type 2. All patients had followed a standard diabetes treatment schedule before recruitment and during the study. The NFJ consumption also increased the insulin excretion (monitored via C-peptide), improved the blood cholesterol status, and reduced the inflammation parameter hs-CRP. The study showed that NFJ can serve as a suitable addition to the diet of patients with diabetes type 2. Further trials with an increased number of participants are warranted to confirm the findings of the present study.

## Figures and Tables

**Table 1:** Basic data of patients, participating in the study.

**No. of patients**	**Age (years)**	**Gender**	**Initial blood** **Glucose level** **(mg/dl)**	**Initial HbA1c-** **blood level** **(**%**)**	**Medicatio** **n** ^**a**^
1	52	F	96	6,3	MF
2	64	M	190	6,0	MF
3	29	M	164	6,9	MF, DPI, IN
4	66	M	100	6,4	MF, DPI
5	62	F	119	6,7	
6	62	F	134	6,5	
7	69	F	138	7,6	
8	61	M	136	7,6	MET, GLP
9	65	M	118	6,3	MET
10	72	M	178	7,0	IN
11	67	M	170	7,1	MET, IN
12	52	M	193	7,1	GLP, IN
13	60	M	179	7,2	IN
14	57	M	135	7,3	MET
15	49	F	158	6,5	DPI, IN
16	43	M	134	6,9	MET
17	77	M	147	7,4	MET
18	60	M	141	6,3	MET
19	62	F	94	5,8	MET
20	69	M	186	7,2	MET, GLP, IN

^a^MET = metformin; DPI= DPP4-inhibitor, GLP1= receptor antagonist; IN= insulin

**Table 2:** Linear regression data of fasting blood sugar values of patients between days 1 and 50 of the intake of NFJ (2ml/kg bw per day).

**No. of patients**	**Intersection with Ordinate at day 0** **(mg/dl)**	**Intersection with Ordinate at day 50** **(mg/dl)**	**Slope** **(mg/dl and day)**
1	103	105	0.032
2	147	160	0.26
3	133	115	-0.36
4	138	116	-0.45
5	141	142	0.014
6	114	101	-0.26
7	122	130	0.15
8	141	126	-0.30
9	125	120	-0.11
10	181	151	-0.61
11	170	164	-0.12
12	162	155	-0.14
13	154	128	-0.52
14	114	106	-0.17
15	136	90	-0.76
17	142	143	0.012
18	145	107	-0.77
19	100	90	-0.21
20	178	126	-1.04
Mean ± SD	139 ± 23	125 ± 22	-0.28 ±0.34
p-value		0.0024^a^	0.002^b^

^a^Calculated from paired t-test (values of day 0 against values of day 50).

^b^Calculated from one sample t-test.

**Table 3:** Changes in the HbA1c (% ) blood level of patients with DT2 during 50 days of NFJ intake (2ml/kg bw per day).

**No. of patients**	**at start**	**after 4 weeks**	**after 8 weeks**
**1**	6.2	6.1	5.6
**2**	6.4	6.6	6.6
**3**	6.9	6.5	6.3
**4**	6.4	6.7	6.6
**5**	6.6	6.6	6.3
**6**	6.9	5.9	5.6
**7**	6.9	6.5	6.8
**8**	7.6	7.0	6.6
**9**	5.4	5.6	5.6
**10**	7.0	7.2	6.7
**11**	7.9	8.0	8.3
**12**	7.2	7.6	7.3
**13**	7.2	7.4	6.9
**14**	7.3	6.9	6.9
**15**	6.5	5.9	5.9
**16**	6.9	6.9	7.4
**17**	7.4	7.3	7.3
**18**	6.3	6.5	6.5
**19**	5.5	5.4	no data
**20**	7.2	6.7	6.3
**Mean (range)**	6.80 (5.5-7.9)	6.67 (5.4-8.0)	6.61 (5.6-8.3)
**SD**	0.64	0.67	0.69
**p-value**		0.17^a^	0.044^b^

^a^Calculated from paired t-test (values at start against values after 4 weeks; N=20).

^b^Calculated from paired t-test (values at start against values after 8 weeks; N=19).

## Data Availability

The data used to support the findings of this study are available from the corresponding author upon request.

## Abbreviations

AMPK: Adenosine monophosphatase kinase
bw: Body weight
CRP: C-reactive protein
DAA: Deacetylasperulosidic acid
DT-1 (2): Diabetes type 1 (2)
GLP-1: Glucagon-like peptide 1
HDL: High density lipoprotein
HbA1c: Glycated hemoglobin
hs-CRP: High sensitive C-reactive protein
LDL: Low density lipoprotein
NFJ: Noni fruit juice
SD: Standard deviation
T2Rs: Mammalian taste receptors.

## Additional Points

*Author's Information*. Petra Algenstaedt is the leading physician of the Department of Endocrinology and Diabetology of the University Clinic Hamburg-Eppendorf and supervisor of the present study. Alexandra Stumpenhagen is physician and doctoral student of Petra Algenstaedt. Johannes Westendorf is professor emeritus of the Institute of Experimental Pharmacology and Toxicology of the University Clinic Hamburg-Eppendorf.
